# Acute Perforated Large Bowel Obstruction Due to Colorectal Malignancy: A Surgical Emergency With Options

**DOI:** 10.7759/cureus.84217

**Published:** 2025-05-16

**Authors:** Melissa Ikizoglu, Michelle Sahagian, Jordan Roy, Peter Rhee

**Affiliations:** 1 Surgery, St. Barnabas Hospital Health System, Bronx, USA; 2 Surgery, New York Institute of Technology College of Osteopathic Medicine, Old Westbury, USA

**Keywords:** colorectal cancer, emergency exploratory laparotomy, hand-sewn anastomosis, large bowel obstruction, large bowel perforation, mbo: malignant bowel obstruction, open colorectal surgery, primary anastomosis, sigmoid colon adenocarcinoma, subtotal colectomy

## Abstract

Acute large bowel obstruction caused by colorectal cancer is a critical surgical emergency, often presenting as a life-threatening condition. This case involves a 71-year-old male who presented to the emergency department with complete large bowel obstruction secondary to a sigmoid mass, raising concern for malignancy. Initial plans were made for decompression and colonoscopy. However, the patient left the hospital against medical advice and subsequently returned two days later with massive abdominal distension and peritonitis. Emergent exploratory celiotomy revealed a septic abdomen with gross contamination due to perforation in the ascending colon near the hepatic flexure, which was not amenable to repair. At a minimum, a subtotal colectomy was required. The patient underwent a single-stage oncologic subtotal colectomy with a hand-sewn ileosigmoid anastomosis and fascial closure. Pathologic evaluation confirmed stage IIb invasive sigmoid adenocarcinoma with negative margins and 0/43 lymph nodes. The patient was discharged on postoperative day nine without complications. For patients presenting with abdominal sepsis from fecal contamination caused by large bowel perforation due to sigmoid malignancy and obstruction, surgical management options vary widely. These range from damage control surgery with multiple subsequent procedures to single-stage resection and anastomosis. This case underscores the importance of individualized, careful decision-making both preoperatively and intraoperatively. It highlights the factors to consider when evaluating the feasibility of single-stage oncologic surgery and the risks associated with performing a primary anastomosis during emergent operations.

## Introduction

Acute large bowel obstruction (LBO) is a severe and often life-threatening condition that frequently serves as the initial presentation of colorectal cancer (CRC). Malignant bowel obstruction occurs in approximately 7-29% of CRC cases, making it a common indication for emergent colorectal surgeries [1,2].

The surgical management of acute LBO with peritonitis varies based on factors such as the patient’s hemodynamic stability, bowel condition, and the presence of peritoneal contamination or sepsis [3]. Options range from damage control surgery requiring multiple subsequent operations to definitive surgical management during the initial operation. In contrast to elective or semi-elective surgery for LBO, where planned one-, two-, or three-stage resections are possible, emergent presentations with acute peritonitis and gross contamination rarely involve a single-stage resection and anastomosis [4]. Instead, colostomy is often considered the safer, more conservative option due to the heightened risk of anastomotic leakage caused by severe inflammation and infection.

However, primary anastomosis offers significant long-term benefits, including improved quality of life. It avoids the challenges associated with ileostomy, such as fluid losses - a potentially fatal complication in elderly patients requiring multiple surgeries. Additionally, ileostomies in this context are rarely reversed, further impacting the quality of life [3-5].

Notably, while both colostomy and primary anastomosis have a similar mortality rate of approximately 10%, the morbidity associated with stoma creation (16%) is higher than the risk of leakage from primary anastomosis (4-6%) [6]. Hospital stays are also significantly shorter for patients undergoing primary anastomosis (average two weeks) compared to those requiring colostomy (average one month). These factors underscore the importance of individualized decision-making, particularly by experienced surgeons who must carefully balance the risks and benefits of each approach.

In this case, the decision between primary anastomosis and ostomy was carefully weighed, and based on intraoperative findings and clinical judgment, a primary anastomosis was performed. This report examines the decision-making process and outcomes associated with single-stage oncologic surgery with primary anastomosis versus ileostomy following subtotal colectomy in a patient presenting with malignant LBO and peritonitis. It highlights the complexities of managing such cases and emphasizes the need for tailored care based on each patient’s unique clinical scenario.

## Case presentation

A 71-year-old man presented to the emergency department (ED) with a two-week history of constipation, decreased appetite, and diffuse colicky abdominal pain. He had not had a bowel movement for five days despite using multiple over-the-counter laxatives. He also reported a 30-pound weight loss over the past year. His medical history included hypertension and hyperlipidemia, with no prior abdominal surgeries.

**Figure 1:**
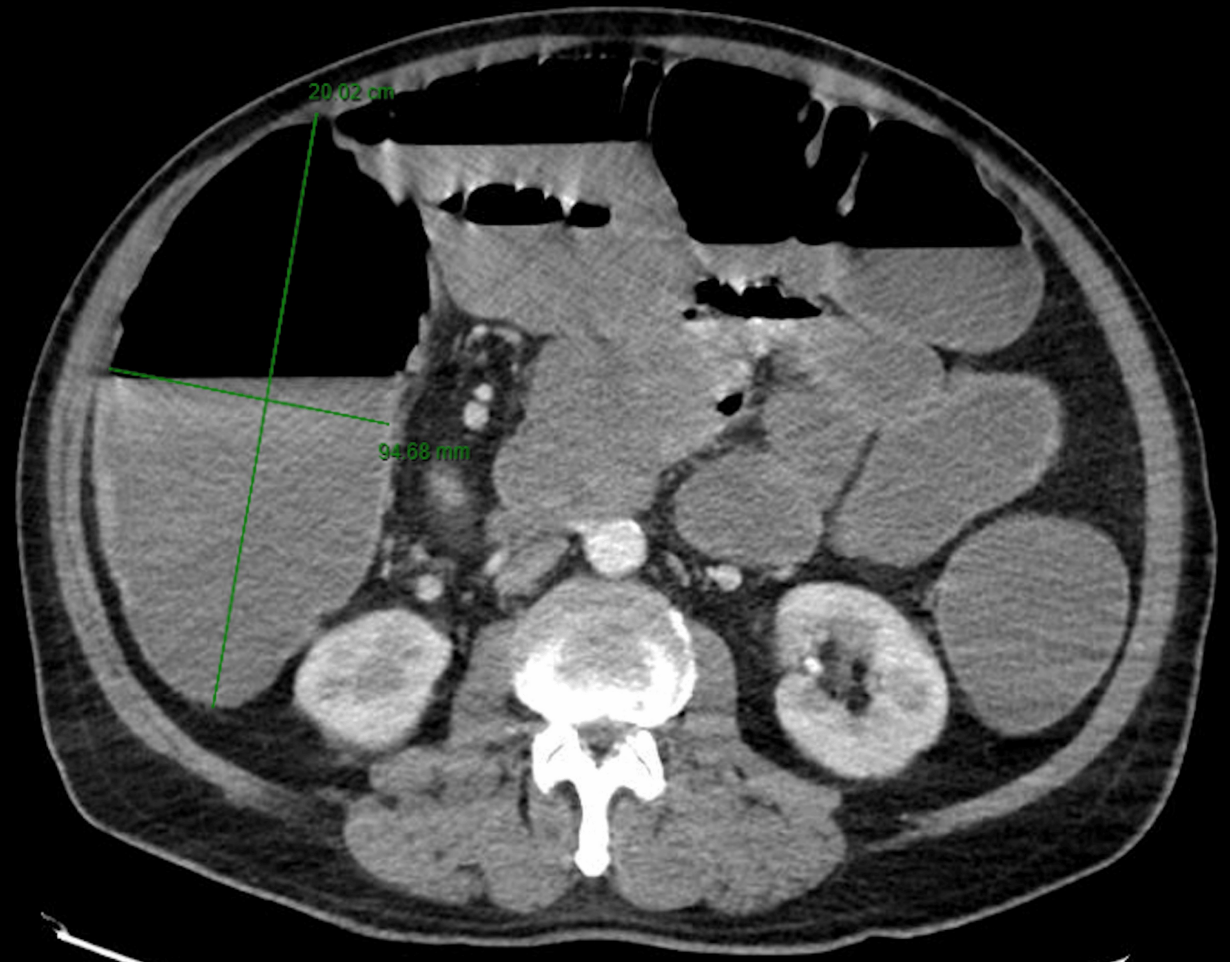
Computerized tomography of the abdomen. The image showed small and large bowel dilation. The right colon measured 20 x 9.5 cm (green linear markers).

**Figure 2:**
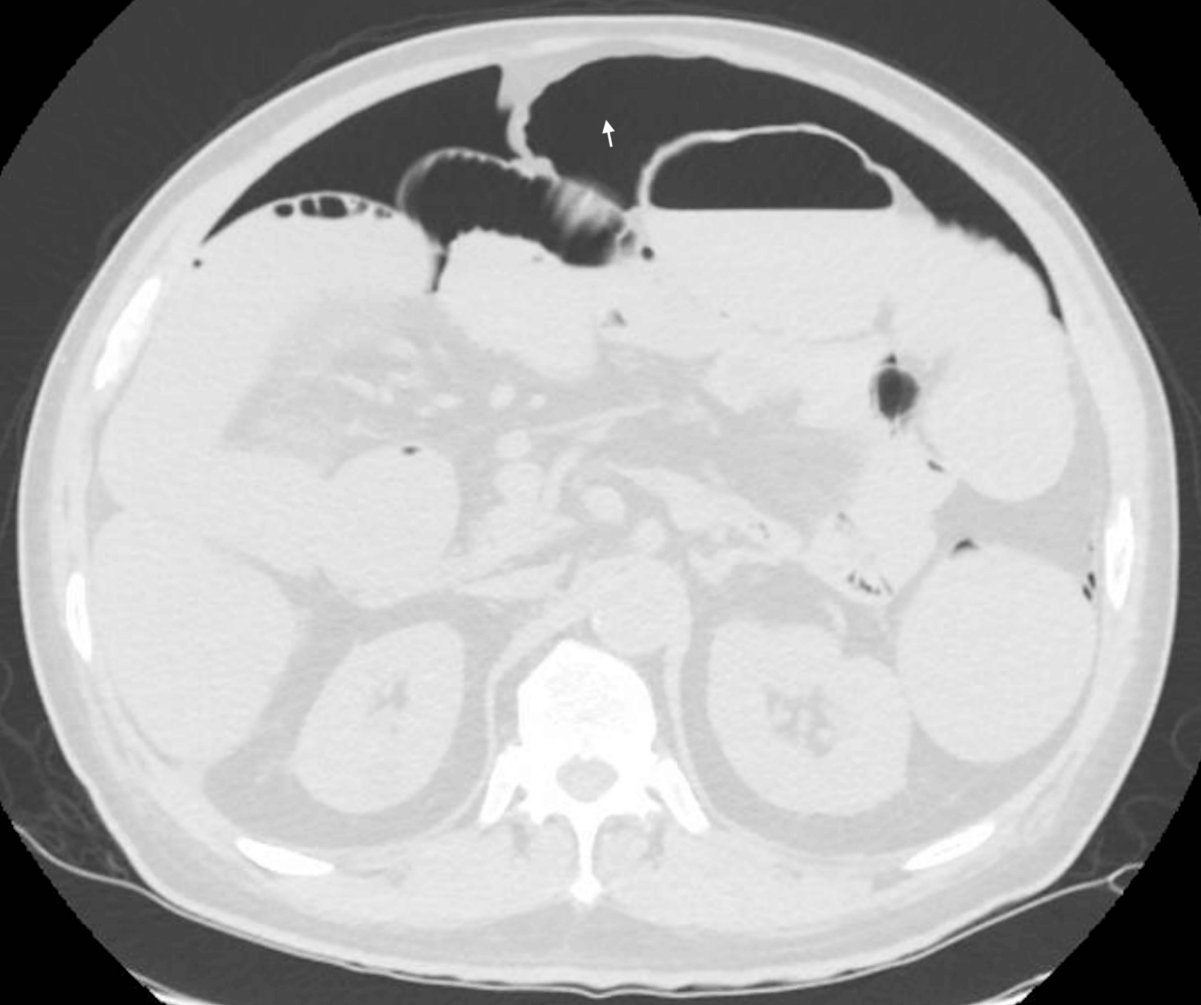
Repeat computerized tomography of the abdomen showed free air (arrow).

On examination, the patient had severe abdominal distension. A computed tomography (CT) scan revealed marked colonic distension with a transition point in the sigmoid colon (Figure 1). Conservative management, including nasogastric decompression, was offered but declined. Plans for colonoscopy and possible stenting were made. However, the patient left the hospital against medical advice. Two days later, he returned with worsening abdominal pain, distress, marked distension, and tenderness. A repeat CT scan revealed a massive pneumoperitoneum, prompting emergent exploratory celiotomy (Figure 2).

Exploration revealed a rectosigmoid mass causing massive dilation of the small and large bowels. An 8 mm perforation was identified near the hepatic flexure in the ascending colon, resulting in severe contamination and abdominal sepsis. No peritoneal studding or metastatic nodules were observed.

Given the extensive contamination and the unsalvageable condition of the ascending colon, a subtotal colectomy was performed. Approximately 12 feet of colon filled with fluid and stool were removed. Due to the extreme colonic dilation (up to 15 cm in the cecum), visualization of the mesentery was challenging. A LigaSure device (Boulder, CO: Covidien) was used to expedite mesenteric resection. The terminal ileum and sigmoid colon were transected with a GIA stapler (Mansfield, MA: Covidien), ensuring a 10 cm margin beyond the rectosigmoid mass for adequate oncologic resection. The mesentery and omentum were resected to optimize lymph node retrieval. Before performing a hand-sewn ileosigmoid anastomosis, fecal contents and concretions were removed from the bowel.

The anastomosis was constructed in two layers using 4-0 absorbable monofilament sutures. The abdominal cavity was thoroughly irrigated, and a Jackson-Pratt drain was placed in the pelvis. The fascia was closed with interrupted 0-Vicryl sutures, and the umbilicus was reapproximated with nylon sutures. Sleeper sutures were placed for planned delayed primary closure. The patient’s systolic blood pressure remained stable (>100 mmHg) throughout the procedure. Intraoperative resuscitation included 4 L of crystalloids, 1 L of colloids, and occasional bolus vasopressor support.

Postoperatively, the patient experienced minimal complications. During his hospital stay, his serum creatinine peaked at 1.5 mg/dL, and he required a transfusion of 2 units of packed red blood cells. He was discharged on postoperative day nine. At his follow-up, his surgical wound had healed well, and he reported improving diarrhea.

Pathology confirmed stage IIb (pT4a, pN0, cM0) invasive adenocarcinoma of the sigmoid colon with TP53, APC, and K-ras mutations. Surgical margins were negative, and 43 lymph nodes were retrieved, all without metastatic involvement. The patient was referred to the tumor board, where postoperative circulating tumor DNA (ctDNA) testing (Guardant Reveal) was performed and found to be negative. Based on his age, tumor stage, and negative ctDNA results, adjuvant chemotherapy was discussed but declined by the patient. He opted for surveillance, with follow-up recommendations including ctDNA testing every three months, CT scans every six months for two years, and a colonoscopy in one year.

## Discussion

Management of malignant large bowel obstruction with perforation

In malignant LBO with perforation, the decision between performing a primary anastomosis or creating a colostomy following subtotal colectomy is influenced by numerous factors that significantly affect patient outcomes. For unstable patients with minimal response to resuscitation, the most conservative approach is often damage control surgery [7]. In cases requiring massive resuscitation and high-dose vasopressor support to maintain hemodynamic stability, immediate stabilization is prioritized. Contamination control through resection, followed by an abbreviated operation, is typically performed to allow for further resuscitation and ventilatory support in the intensive care unit. However, resection alone leaves patients with a definitive small bowel obstruction, potentially leading to significant complications.

Patients undergoing damage control surgery often require multiple subsequent operations, increasing their overall burden of illness, and gravely unstable patients usually need at least an ileostomy [4,8]. Repeated returns to the operating room may result in complications such as a frozen abdomen, necessitating skin grafting for an open abdomen, and an increased risk of a large ventral hernia. Fluid losses from an open abdomen can exacerbate the need for vasopressor support and contribute to respiratory failure, renal failure, and, ultimately, multi-organ failure.

For patients with peritonitis and septic abdomen, resection with ileostomy is a common treatment option. However, this approach frequently requires additional surgeries, and ileostomy reversal is rarely performed, particularly in elderly or frail patients [1,4,9]. Managing an ileostomy can be challenging in these populations due to fluid and electrolyte imbalances, which can be life-threatening. Conversely, if a patient can be stabilized during the initial operation, performing a one-stage oncologic resection with primary anastomosis can reduce the need for future procedures [10]. Still, this approach requires careful consideration of the extent of contamination, the patient’s hemodynamic status, comorbidities, and intraoperative findings.

Primary anastomosis versus stoma creation

Primary anastomosis is generally reserved for patients who are hemodynamically stable and have limited contamination due to higher risks of anastomotic leakage in emergency surgeries involving perforation or peritonitis [11]. Leakage rates in such cases can be up to three times higher than in elective settings. Despite these risks, its advantages include preserved bowel continuity, improved postoperative quality of life, and avoidance of stoma-related challenges [9]. When intraoperative conditions are appropriate, a one-stage resection with anastomosis does not significantly increase morbidity or mortality compared to traditional staged procedures, even in cases with emergency colorectal surgeries with fecal contamination [12,13]. One similar case showed that an ileo-transverse anastomosis would be feasible without postoperative complications in the setting of an emergency right hemicolectomy secondary to a malignancy with an impending cecal perforation [14]. Additionally, a single-stage approach has been associated with lower mortality rates than Hartmann’s procedure, which carries a high risk of complications, and stoma reversal is unlikely.

A protective diverting ostomy may be considered when the risk of anastomotic leakage is high, as it diverts fecal flow and reduces septic complications [5,15]. While potentially beneficial for obstructed left-sided colon cancers with severe bowel dilation or high perforation risk, a diverting ostomy carries its own burdens. Stoma reversal can be complicated and is particularly risky in elderly patients with cancer, who may already face significant morbidity and mortality from additional surgeries [16,17]. For those requiring adjuvant chemotherapy, proximal diversion can mitigate the risks of anastomotic leakage, as infections may delay or preclude chemotherapy.

Institutional resources and social circumstances also influence the choice of surgical approach. In this case, the surgery was performed at an urban public hospital serving a low-income population with limited access to follow-up care. These factors must be weighed when determining the feasibility of long-term stoma management or additional surgeries. For patients with obstructive CRC, untreated obstruction can lead to perforation and emergent surgery. Preoperative management typically includes resuscitation and empiric antibiotics to manage sepsis [11]. While endoscopic stenting is sometimes used as a bridge to surgery for non-perforated obstructions, it is contraindicated in the setting of perforation due to the risk of further contamination [18].

Case analysis

In this patient’s surgery, a primary hand-sewn anastomosis was chosen based on favorable intraoperative conditions and the patient’s response to resuscitation. The dynamic decision-making process accounted for the extent of contamination, patient stability, comorbidities, and the patient’s desire to avoid a stoma. Despite the inherent risks, the patient’s postoperative course was uneventful, aside from a mild, transient rise in serum creatinine. This successful outcome demonstrates that a primary anastomosis can be a viable option even in the presence of abdominal sepsis caused by a perforated LBO due to CRC.

Proximal diversion was not performed in this case due to limited supporting evidence and the specific risks associated with elderly patients. Avoiding a stoma aligned with the patient’s preference and led to a successful outcome. This case highlights that while colostomy is often the default approach for malignant LBO with perforation, it is not always necessary. Individualized surgical planning, taking into account the patient's stability, intraoperative findings, and socio-environmental factors, can optimize outcomes.

## Conclusions

This case underscores the complexities involved in managing acute malignant bowel obstruction with perforation. It demonstrates that primary anastomosis can be a feasible option for an elderly patient with fecal contamination and abdominal sepsis caused by obstructing colon cancer. It is also possible to achieve oncologic resection, even in such emergencies. Although colostomy is commonly perceived as the most conservative approach, it should not be regarded as the only choice. When patient stability, intraoperative findings, and overall clinical context are favorable, primary anastomosis can be pursued safely. Ultimately, the decision between diversion and primary anastomosis must be individualized, balancing surgical risks with patient-specific medical and socioeconomic circumstances. By doing so, surgeons can optimize outcomes and enhance the quality of care in these challenging scenarios.
